# Observation on the clinical efficacy of external ventricular drain combined with intraventricular urokinase injection and intravenous piracetam in the treatment of intraventricular hemorrhage

**DOI:** 10.12669/pjms.38.5.4820

**Published:** 2022

**Authors:** Jing Cui, Xin-lei Ma, Jian-zhou Tong, Min Shu

**Affiliations:** 1 Jing Cui, Department of Neurology, Baoding First Central Hospital, Baoding, 071000, Hebei, P.R. China; 2 Xin-lei Ma, Department of Neurology, Baoding First Central Hospital, Baoding, 071000, Hebei, P.R. China; 3 Jian-zhou Tong, Department of Neurology, Baoding First Central Hospital, Baoding, 071000, Hebei, P.R. China; 4 Min Shu, Department of Neurology, Baoding First Central Hospital, Baoding, 071000, Hebei, P.R. China

**Keywords:** External ventricular drain, Intraventricular urokinase injection, Piracetam, Intraventricular hemorrhage

## Abstract

**Objective:**

To observe the clinical efficacy of external ventricular drain combined with intraventricular urokinase injection and intravenous piracetam in the treatment of intraventricular hemorrhage.

**Methods:**

A randomized controlled trial was used in this study conducted at Baoding First Central Hospital, China from January 2017 to December 2019. Sixty patients with intraventricular hemorrhage were randomly divided into two groups. Patients in the control group were treated with external ventricular drain, while patients in the experimental group were given intraventricular urokinase injection and intravenous piracetam on the basis of the control group. The incidence of adverse drug reactions, hospitalization time, hematoma elimination time, and drainage tube removal time in two groups were compared and analyzed including the cerebrospinal fluid protein content, changes in GCS score, neurological function recovery (ADL score), and Glasgow outcome scale (GOS) of the two groups after treatment.

**Results:**

The hematoma elimination time, drainage tube removal time and hospitalization time of the experimental group were shorter than those of the control group, with a statistically significant difference (P<0.05). After treatment, compared with the control group, the protein content of cerebrospinal fluid in the experimental group decreased more significantly (P=0.00), the GCS score was higher (P=0.00), the overall good rate of neurological function was higher (P=0.04), while the rate of good prognosis was higher (P=0.03). Within one month of treatment, the incidence of surgical complications in experimental group was significantly lower than that in control group (P=0.04).

**Conclusions:**

External ventricular drain combined with intraventricular urokinase injection and intravenous piracetam is an effective method for the treatment of intraventricular hemorrhage, which is worthy of clinical promotion.

## INTRODUCTION

Cerebral hemorrhage, a very common clinical acute cerebrovascular disease, is characterized by rapid onset, fast progress, critical condition, high disability rate and high mortality.^1^ One of the more serious types of cerebral hemorrhage is intraventricular hemorrhage, which has been reported in the literature to have a mortality rate of more than 50%.^2^ In case of intraventricular hemorrhage, serious consequences will be caused. Specifically, the obstruction of cerebrospinal fluid circulation may be caused, leading to acute obstructive hydrocephalus.^3^

Endoscopic hematoma removal and external ventricular drain are currently the preferred methods for the treatment of patients with intraventricular hemorrhage.^4^ It was considered in the study of Mei et al.^5^ that endoscopic surgery has a direct effect on hematoma removal, but it will further increase the relative brain parenchymal damage. External ventricular drain, by contrast, boasts the advantages of less damage, simple and rapid operation, etc. It can also quickly alleviate intracranial hypertension and drainage of intraventricular hematocele, thereby relieving hydrocephalus. However, the ventricular drainage tube is easily obstructed by blood clots, resulting in poor drainage and reduced clearance of intraventricular hematocele.

Intraventricular fibrinolysis (IVF), represented by urokinase, can maintain the patency of cerebrospinal fluid access and ventricular catheter, thus removing hematoma faster, reducing intracranial pressure better, promoting the recovery of cerebrospinal fluid circulation, and reducing the contact time between catheter and subarachnoid membrane.^6^ Piracetam is a positive allosteric modulator of the α-amino-3-hydroxy-5-methyl-4-isoxazolepropionic acid receptor, which is often used to treat cognitive disorders, and may promotes the recovery of the damaged brain.^7^ Functional protection of nerve cells also plays a positive role in the recovery of patients with intraventricular hemorrhage. In this research, patients with intraventricular hemorrhage were treated with external ventricular drain combined with intraventricular urokinase injection and piracetam. The specific details are reported as follows:

## METHODS

A randomized controlled trial was used in this study. A total of 60 patients with intraventricular hemorrhage admitted to Baoding First Central Hospital from January 2017 to December 2019 were included in the study according to the random number table method, and were randomly divided into experimental group and control group according to the principle of random draw, with 30 patients in each group.

### Ethical approval:

The study was approved by the Institutional Ethics Committee of Baoding First Central Hospital on March 21, 2017 (No.: 2041ZF101), and written informed consent was obtained from all participants or their families.

### Inclusion criteria:

- Patients who met the diagnostic criteria for intraventricular hemorrhage by CT or angiography^8^;
- Patients with spontaneous intraventricular hemorrhage or periventricular tissue hemorrhage rupturing into the ventricle;
- Patients younger than 60 years old;
- Patients who underwent external ventricular drain within 24h of onset;
- Patients with obvious neurological symptoms and Glasgow coma scale (GCS) ≤ 8;
- Patients or their immediate family members voluntarily participated in the study;
- Patients without allergy to the drugs needed for the study.

### Exclusion criteria:

- Patients with serious diseases in important organs such as heart, liver, kidney;
- Patients with active cerebral hemorrhage;
- Patients with intraventricular hemorrhage caused by cerebral aneurysms and cerebrovascular malformations;
- Patients who have recently taken anticoagulants;
- Patients with coagulation dysfunction. Patients in the experimental group were given intraventricular urokinase injections and piracetams, while patients in the control group were treated with external ventricular drain alone.

Among all the patients, 17 males and 13 females were grouped into the experimental group, aged 32-58 years old, with an average of 42.50±8.07 years old. 19 males and 11 females were grouped into the control group, aged 26-59 years old, with an average of 42.37±8.31 years old. There was no significant difference in general data between the two groups (P>0.05), which was comparable between the two groups (Table-I).

**Table I:** Comparative analysis of general data between the two groups (χ¯±S) n=30.

Indicators	Experimental group	Control group	t/χ^2^	p
Male (number of cases, %)	17 (57%)	19 (63%)	0.28	0.60
Age (years old)	42.50±8.07	42.37±8.31	0.07	0.94
Primary hemorrhage site				
Intraventricular hemorrhage (number of cases, %)	13 (43.3%)	10 (33.3%)	0.63	0.43
Paraventricular hemorrhage rupturing into the ventricle (number of cases, %)	12 (40%)	14 (46.7%)	0.27	0.60
Basal ganglia hemorrhage rupturing into the ventricle (number of cases, %)	3 (10%)	4 (13.3%)	0.16	0.69
Cerebellar hemorrhage rupturing into the ventricle (number of cases, %)	2 (6.7%)	2 (6.7%)	0.00	1.00
Hemorrhage volume	17.93±2.36	18.21±3.01	0.72	0.28
KPS score	46.56±4.82	46.33±5.07	0.21	0.84
GCS score	6.28±1.01	6.05±0.76	1.15	0.25

p>0.05,

*KPS means Karnofsky Performance Status, GCS means Glasgow Coma Scale.

### Treatment methods:

Patients in the control group were treated with external ventricular drain (EVD). Patients underwent vital signs monitoring and neurological assessments, as well as supportive therapies such as oxygen inhalation after admission. Unilateral or bilateral EVD was decided according to the patient’s age, blood pressure, state of consciousness and the results of head CT images. Patients were placed in supine positions, and frontal puncture of one or both ventricles was performed. The puncture point was 2.5cm in front of the coronal suture and 2.5cm beside the midline. The direction of the puncture was parallel to the sagittal plane and aligned with the line of the external auditory canal with a depth less than 6cm. The puncture was successful when the needle core was removed and bleeding cerebrospinal fluid was seen. The F12 silicone drainage tube was indwelled and properly fixed. The drainage volume was recorded daily and the patency of the drainage tube was observed. A Head CT scan was performed dynamically to determine the hemorrhage volume and the location of the drainage tube until the ventricular drainage tube was removed.

In addition to treated with EVD, patients in the experimental group were also given indoor urokinase injection and piracetam injection intravenous infusion. Specific methods: 4g piracetam injection, intravenous drip 250ml of glucose or normal saline, qd. Intraventricular urokinase injection method: From the 1st day after the EVD surgery, 30,000 U of urokinase + 5ml of normal saline were injected into each ventricular drainage tube every day. After the injection, the tube was closed for two hour and then the drainage tube was opened for 24 hours until the ventricular drainage tube was pulled out. All patients were followed up for six months.

### Observation indicators:

1) The hospitalization time, hematoma elimination time, and drainage tube removal time were compared and analyzed between the two groups. Criteria for drainage tube removal^9^: Head CT scan shows a significant decrease or disappearance of intraventricular hematocele, without hematocele or ventricular dilatation in the third and fourth ventricles; Repeated attempts to clamp the drainage tube, and patients have no symptoms of consciousness disorder or intracranial hypertension

The differences in CSF protein levels and GCS scores between the two groups at two and four weeks after treatment were compared and analyzed; Judgment of treatment effect: The recovery of the living ability of the two groups of patients after three months of treatment was observed. All patients were graded according to the neurological recovery score (ADL rating scale) after treatment^10^: Grade I: completely normal life, Grade II: able to basically take care of themselves in life, Grade III: living in need of help from others, Grade IV: conscious but unable to take care of themselves, Grade V: death. The overall good rate = Grade I + Grade II + Grade III. Judgment of prognosis: After six months of treatment, the Glasgow Outcome Scale (GOS)^11^ was used to evaluate the prognosis of the patient: one point: death; two points: vegetative state; three points: severe disability; four points: moderate disability; five points: good recovery, or mild disability. A GOS score of 4-5 indicates a good prognosis. The proportion of patients with a good prognosis between the two groups was compared and analyzed. *Surgical complications:* The incidence of surgical complications within one month of treatment was compared and analyzed between the two groups.

### Statistical analysis:

All the data were statistically analyzed by SPSS 20.0 software, and the measurement data were expressed as (X̄ ± s). Two independent sample t-test was used for inter-group data analysis, paired t test was used for intra-group data analysis, and χ^2^ was adopted for rate comparison. P<0.05 indicates a statistically significant difference.

## RESULTS

The hospitalization time, hematoma elimination time and ventricular drainage removal time between the two groups were compared and analyzed (Table-II). It suggested that the hematoma elimination time, ventricular drainage removal time and hospitalization time in the experimental group were shorter than those in the control group, with a statistically significant difference (P<0.05).

**Table II:** Comparison of hospitalization time, hematoma elimination time and ventricular drainage removal time between the two groups (χ¯±S) n=30.

Group	Hematoma elimination time (h)	Ventricular drainage removal time (h)	Hospitalization time (d)
Experimental group	43.53±11.28	82.76±13.74	22.75±7.64
Control group	52.61±13.32	97.83±16.92	27.60±8.07
t	2.85	3.78	2.39
p	0.01	0.00	0.02

P<0.05.

The protein content in the cerebrospinal fluid of the experimental group decreased obviously after two weeks and four weeks of treatment, with a statistically significant difference (P=0.00). After treatment, the GCS scores of the two groups were significantly improved, while the GCS scores of the experimental group were significantly higher than that of the control group after two and four weeks of treatment, with a statistically significant difference (P=0.00). (Table-III).

**Table III:** Comparative analysis of cerebrospinal fluid protein and GCS scores of the two groups after treatment (χ¯±S) n=30.

	Cerebrospinal fluid protein level (g/L)				GCS score			

	Experimental group	Control group	t	p	Experimental group	Control group	t	p
2 weeks	0.77±0.03	0.85±0.04	8.76	0.00	7.06±0.21	6.47±0.32	8.44	0.00
4 weeks	0.53±0.01	0.72±0.03	32.91	0.00	8.03±0.30	7.02±0.24	14.40	0.00
t	41.60	14.24			14.51	7.53		
p	0.00	0.00			0.00	0.00		

P<0.05.

The recovery of neurological function of the two groups after 3 months of treatment was compared and analyzed, indicating that the total excellent and good rate of neurological function recovery of the experimental group was higher than that of the control group, with a statistically significant difference (P=0.04) (Table-IV).

**Table IV:** Comparative analysis of ADL scores between the two groups after treatment (χ¯±S) n=30.

Group	Grade I	Grade II	Grade III	Grade IV	Grade V	Total excellent and good rate (%)
Experimental group	4	7	10	7	2	21 (70%)
Control group	3	4	6	14	3	13 (43.3%)
χ^2^						4.34
p						0.04

p<0.05.

After six months of treatment, the GOS score of the two groups showed a good prognosis rate of 66.7% in the experimental group, which was significantly better than 40% in the control group, with a statistically significant difference (P=0.03). (Table-V).Within one month of treatment, the incidence of surgical complications in the experimental group was 13.3%, which was significantly lower than that 36.7% in the control group, with a statistically significant difference (P=0.04). (Table-VI).

**Table V:** Comparative analysis of GOS scores between the two groups after treatment (χ¯±S) n=30.

Group	1 point	2 points	3 points	4 points	5 points	Good prognosis rate (%)
Experimental group	2	4	4	13	7	20 (66.7%)
Control group	3	9	6	7	5	12 (40%)
χ^2^						4.28
p						0.03

p<0.05.

**Table VI:** Comparative analysis of the incidence of complications between the two groups (χ¯±S) n=30.

Group	Intracranial infection	Hydrocephalus	Rehemorrhage	Total incidence (%)
Experimental group	1	2	1	4 (13.3%)
Control group	3	4	4	11 (36.7%)
χ^2^				4.36
p				0.04

P<0.05.

## DISCUSSION

Intraventricular hemorrhage (IVH), as a clinically common severe cerebrovascular disease, is characterized by rapid onset, high mortality and disability.^12^ It is one of the most challenging diseases for neurosurgeons. Despite the continuous improvement of treatment methods for IVH in recent years, the success rate is low and the complication rate is high.^13^ In the late stage, complications such as respiratory circulation disorder, cerebral hernia and decerebral rigidity may occur, and the obstruction of cerebrospinal fluid circulation to varying degrees may aggravate brain injury, leading to a very high mortality rate with conservative treatment.^14^ The hematoma formed will cause obstruction to the cerebrospinal fluid circulatory pathway. Hydrocephalus and a variety of complications may result from this.^15^ Consequently, the key to the treatment of IVH is to relieve the compression of intracerebral parenchyma by intraventricular hematoma and protect the brain tissue from further injury to the maximum extent.^16^ It has been shown in recent studies^17^ that in the case of intracerebral hemorrhage combined with IVH, EVD may be a promising method to improve blood clearance rate, boasting advantages such as less damage to brain tissue, simple operation, short duration and so on. However, adverse effects such as cerebral ventricle easily blocked by blood clots, poor drainage, reduced intraventricular hematocele clearance will also follow.

Urokinase, a plasminogen activator, boasts various benefits such as rapid dissolution of intracerebral hematoma, better reduction of secondary brain damage in patients with intracerebral intraventricular hematoma, effective reduction of intracranial pressure and fewer side effects.^18^ Piracetam plays a protective role against cerebral hypoxia injury, promotes the recovery of the damaged brain, and has no adverse reaction or dependence on psychotropic drugs.^19^ Results of a meta-analysis showed that, compared with EVD alone, EVD combined with urokinase improved the survival and prognosis of patients with IVH.^20^ In addition, an earlier study had found that, there were fewer deaths in piracetam-treated patients in those patients with primary hemorrhagic stroke.^21^ In our research, the overall good rate of neurological function recovery, as well as the rate of good prognosis in patients of experimental group, were higher than those of the control group, while the incidence of surgical complications was lower than that in the control group. These results were similar to those of previous studies. In our study, the hematoma elimination disadvantages of such a treatment regimen intime, drainage tube removal time and hospitalization time of experimental group were shorter than those of the control group. After treatment, the protein content of the cerebrospinal fluid in the experimental group decreased more obviously than that in the control group, and the GCS score was significantly higher than that in the control group. These results were similar to those of Yang et al.^22^

### Limitations of this study:

The sample size of this study was small and the follow-up time was short. In addition, only patients with simple ventricular drainage were set as the control group, and the application of different fibrinolytic drugs was not compared to clarify whether there are different therapeutic effects between different drugs. In response to this, proactive measures will be taken in the future to further increase the sample size, design a more reasonable study protocol, include different drugs for comparative study and extend the follow-up time, so as to evaluate the advantages and a more detailed and objective manner. In addition, more rational drugs are being sought to benefit patients with intraventricular hemorrhage.

## CONCLUSION

EVD combined with intraventricular urokinase injection and intravenous piracetam is an effective method for the treatment of intraventricular hemorrhage. With such a regimen, the hospitalization time, hematoma elimination time, drainage tube removal time, as well as the incidence of surgical complications can be reduced, the neurological function can be recovered to a certain extent, and the prognosis of patients can be improved.

### Authors’ Contributions:

**JC&XLM:** Designed this study and prepared this manuscript, and are responsible and accountable for the accuracy or integrity of the work.

**JZT:** Collected and analyzed clinical data.

**MS:** Significantly revised this manuscript.
